# Music engages the phasic dopaminergic D1-receptor system in humans: a PET-fMRI study using [^11^C]SCH23390

**DOI:** 10.1007/s00259-026-07837-y

**Published:** 2026-04-14

**Authors:** Thomas Hans Fritz, Johanna Girbardt, Michael Rullmann, Henryk Barthel, Franziska Zientek, Georg-Alexander Becker, Marianne Patt, Max Archibald Montgomery, Arno Villringer, Swen Hesse, Osama Sabri

**Affiliations:** 1 Department of Neurology, Max Planck Institute for Human Cognitive and Brain Sciences, Stephanstraße 1a, 04103 Leipzig, Germany; 2 Institute for Psychoacoustics and Electronic Music (IPEM), University of Gent, Technicum Blok 2, Sint-Pietersnieuwstraat 41, 9000 Ghent, Belgium; 3 Institute for Medical Informatics, Statistics and Epidemiology, Leipzig University, Härtelstr. 16-18, 04107 Leipzig, Germany; 4 Department of Nuclear Medicine, University of Leipzig, Liebigstraße 18, 04103 Leipzig, Germany

**Keywords:** Dopamine, D1, PET, FMRI, Music, Valence, Arousal, Striatum, [^11^C]SCH23390

## Abstract

**Introduction:**

Music listening reliably induces arousal in the listener, however its effect on dopaminergic transmission is not yet fully explored.

**Material and methods:**

We investigated the responsiveness of the striatal dopamine D1 receptor (D1R) system, which is preferentially engaged by phasic dopaminergic signals, using simultaneous PET-fMRI and the selective D1R radioligand [^11^C]SCH23390. Fifteen neurotypical participants were scanned during silence before listening to a music piece in its pleasant and unpleasantly manipulated variation. Striatal binding potential (BP_ND_) was significantly lower when listening to music compared to silence.

**Results:**

A seed-based fMRI analysis revealed higher functional connectivity between the striatum and left dorsolateral pre-frontal cortex (MNI: x = -43, y = 24, z = 24) during pleasant music in relation to unpleasant music. An exploratory whole-brain D1R PET analysis further identified increased insular BP_ND_ (MNI: x = -33, y = 1.5, z = 16.5; T = 5.58) during pleasant relative to unpleasant music. Comparison of BP_ND_ and fMRI beta estimates changes within that prefrontal cluster showed a moderate correlation between the pleasant and unpleasant music condition (R = -0.52, *p* = 0.046), but no correlation for the silent conditions (R = 0.24, *p* = 0.42). Changes in ratings of musical pleasantness moderately correlated with fMRI changes in the prefrontal cluster, but not with changes in BP_ND_.

**Conclusion:**

Our results demonstrate that music irrespective of valence engages the D1R system, indicating that the inherently phasic nature of music can drive D1R-related responses. Furthermore, our results demonstrate valence effects in functional connectivity between the striatum and the left dorsolateral prefrontal cortex when listening to music with varying pleasantness.

## Introduction

Listening to music can have an arousing effect in humans and quite unarguably such emotional and physical arousal effects are among the major incentives for listening to music in the first place. In affective models, pleasantness and arousal are often described as having a V-shaped relationship, such that both highly pleasant and highly unpleasant stimuli are associated with increased arousal. At the same time, empirical work indicates that this relationship is not fixed but varies as a function of individual differences, musical expertise, and contextual factors [1, 2].

Dopamine (DA) has been shown to be one of the primary neurotransmitters in the brain reward circuit [3–5]. Mesolimbic DA modulates incentive salience or ‘wanting’ of rewards [6]. While it has become apparent that the DA D2 receptor (D2R) system plays an important role in the mediation of musical experience [7] and recent work could also provide evidence for a prominent role of μ-opioid receptors in the response to pleasurable music [8], the role of D1 receptors (D1R) in the mediation of our response to music remains less clear. Importantly, dopaminergic receptor systems have been shown to subserve partially dissociable functions, with D1 receptor signaling being preferentially associated with phasic dopamine responses related to motivational salience and action invigoration, whereas D2 receptor signaling has been more strongly linked to tonic dopamine activity and reward learning processes, and μ-opioid receptor activity to hedonic pleasure [9]. D1R has been implicated in arousal modulation in the striatum and the nucleus accumbens (NAc), both D1- and D2-expressing medium spiny neurons of the NAc have been shown to drive both reward and aversion according to the respective neuronal stimulation pattern [10].

The striatum is crucial for emotional responses to music, with pleasurable music increasing [^11^C]carfentanil binding in “hedonic hotspots” like the ventral striatum/NAc, correlating with chills [8]. That the striatum is especially relevant to mediating an emotional response to music is further substantiated by findings showing that it plays a role in musical enjoyment, but not in participants with musical anhedonia [11]. The NAc was also shown to predict the amount of money spent for the acquisition of musical excerpts in an auction paradigm [12]. Using [^11^C]raclopride PET scanning to assess D2R binding, endogenous DA release in the striatum at peak emotional arousal during music listening was measured [7]. Because the striatum contains high densities of both D1Rs and D2Rs [13], it is reasonable to assume that it serves as a crucial node for integrating DA activity acting on both receptor classes. Although PET-based measures of dopamine receptor binding do not capture moment-to-moment subjective experience, multimodal and pharmacological studies provide evidence that dopaminergic transmission is closely related to music-evoked arousal and reward. Combined PET–fMRI studies have demonstrated striatal dopamine release during music listening in relation to emotional engagement and reward anticipation [7, 12], and pharmacological manipulation of dopamine has been shown to modulate subjective pleasure and motivation in response to music [14]. These findings support a mechanistic link between PET-measured dopaminergic engagement and subjective music-evoked arousal and reward.

The challenge of investigating the effect of music on the D1R system is, on the one hand, due to difficulties in developing tracer drugs that selectively bind to D1Rs, while also maintaining good bioavailability throughout the measurement [15]. On the other hand, this challenge is compounded by the difficulty of reliably activating the D1R system using behavioural tasks. Given evidence that D1 receptor signaling is preferentially engaged by phasic dopamine bursts and plays a key role in striatal arousal as well as cognitive–emotional integration [16], the D1R system represents a plausible target for investigating music-evoked arousal (because music is also phasically organized). We therefore sought to examine if the D1R system responds differently during music listening compared to previous findings focusing primarily on the D2R system.

We used [^11^C]SCH23390 to quantify D1R binding, as this radiotracer has been shown to be capable to determine variations in D1R binding in the striatum [17]. The radiotracer [^11^C]SCH23390 has previously been shown to also be suitable for investigating cortical areas, e.g. the prefrontal cortex (PFC) [18, 19], where D1R are more abundant than D2R [20]. As a well-established antagonist radioligand for the D1 receptor family [17, 21, 22], [^11^C]SCH23390 enables reliable mapping of both striatal and extrastriatal D1R distributions in vivo [20, 23]. Given these properties, [^11^C]SCH23390 provides a suitable tool to probe D1R responses during music listening in the present study.

Based on the evidence outlined above regarding music-evoked arousal, dopaminergic signaling, and the specific role of D1 receptors in phasic dopamine transmission, the present study aimed to systematically test the following hypotheses: Such knowledge is crucial for understanding D1R as a potential intervention target in neuropsychiatric conditions such as depression, attention-deficit/hyperactivity disorder (ADHD), schizophrenia, and Parkinson’s disease (PD) [24–29]. Specifically, we hypothesized that 1. music listening engages the phasic-sensitive D1R system, consistent with evidence linking D1 receptor signaling to phasic dopamine activity and arousal-related processing; 2. the D1R response to music can be reliably measured in the striatum, a key hub for dopaminergic and music-evoked emotional processing and 3. [^11^C]SCH23390 allows the investigation of potential extrastriatal D1R-related responses to music, including valence-dependent effects in cortical and limbic regions implicated in music perception and affect. To guide the analysis of hypothesis (3), we focused on a predefined set of regions of interest, including substantia nigra/ventral tegmental area, ventral and dorsal striatum, orbitofrontal and dorsolateral prefrontal cortex, amygdala, hippocampus, anterior cingulate cortex, and rolandic operculum. These regions have repeatedly been implicated in reward, affective processing, and music-evoked emotions [12, 30–32]. They therefore provided a theoretically motivated target network for probing potential extrastriatal D1R effects during music listening.

## Materials and methods

### Participants

Fifteen (8 females, age 32 ± 13 years) neurotypical, right-handed, non-smoking participants with normal hearing were recruited in Leipzig. Exclusion criteria included cerebral lesions, neuropsychiatric disorders, psychotropic or illicit drug use, MRI contraindications, nicotine abuse, pregnancy or breast-feeding, and more than five years of formal musical training; individuals reporting a strong aversion to music were excluded. For two participants, one of the neutral scans could not be acquired due to technical device failure, resulting in *n* = 13 for analyses requiring both neutral conditions.

### Stimuli

Music pieces and their unpleasant manipulated counterparts were chosen via a pre-study with 51 volunteers. Unpleasant versions were continually dissonant, consistent with prior research [33–35]. Unaltered stimuli were instrumental major-minor tonal music from the last four centuries. Dissonant versions were created using Ableton Live (Ableton AG, Berlin, Germany) by simultaneously playing the music in original pitch, a semitone higher, and a tritone lower (e.g. [32, 35]). Total music duration was 60 min (15 pieces). In line with previous studies [7, 32, 36], all participants listened to the same set of acoustically manipulated musical stimuli, independent of individual musical preferences.

### Procedure/study design

This within-subject challenge study was performed at the Department of Nuclear Medicine, University of Leipzig. Participants gave written informed consent. Each participant underwent 4 scans on 2 days, with 2 scans per day. Neutral condition scans were around 9:00, and music conditions around 15:00. Music conditions (pleasant/unpleasant) were balanced across participants. Participants closed their eyes and stayed awake in the scanner, listening to music via MRI-compatible headphones (VisuaStim, Resonance Technology, Inc., Northridge, CA). The music stimulus started 10 min prior to the start of the PET/fMRI scan to allow neurotransmitter levels to increase and reach a new, elevated quasi–steady state and lasted until 50 min p.i. After each scan, music was rated on a 0–100 pleasantness scale.

### Radiotracer preparation

[^11^C]SCH23390 was prepared from SCH24518 (ABX, Radeberg, Germany) using the captive solvent method [37] with [^11^C]CH3I (MeI Microlab, GE Medical Systems, Waukesha, WI, USA). Specific activity was 3.0 ± 1.0 × 10^5^ GBq/mmol via analytical high-performance liquid chromatography.

### Brain PET/MR imaging

Data were acquired using an integrated PET/MR system (Biograph mMR, Siemens, Erlangen, Germany). Dynamic PET scan were obtained combined with echo planar imaging (EPI) volumes and T1-weighted 3D magnetization-prepared rapid gradient-echo (MPRAGE). A two-point MRI Dixon volumetric interpolated breath-hold examination (VIBE) sequence was acquired for attenuation correction before injection. Participants received a bolus injection of 487 ± 19 MBq [^11^C]SCH23390 followed by a dynamic 90 min PET scan (23 frames: four 0.25 min, four 1 min, five 2 min, five 5 min, five 10 min), which is required to ensure reliable kinetic modeling and accurate estimation of [^11^C]SCH23390 D1R binding potential. The injected mass did not differ between the four scans (neutral 1: 0.09 ± 0.17 μg/kg, pleasant: 0.07 ± 0.11 μg/kg, neutral 2: 0.07 ± 0.14 μg/kg, unpleasant: 0.07 ± 0.13 μg/kg, ANOVA *p* = 0.97).

PET data were motion-corrected with SPM8 (Statistical Parametric Mapping, Wellcome Trust Centre for Neuroimaging, University College London). Individual T1-weighted MPRAGE and motion-corrected PET data were reoriented according to the anterior and posterior commissure line and resliced to 1 mm isotropic voxel size. Parametric images of D1R binding potential (BP_ND_) were generated using PMOD (PMOD Technologies, Zurich, Switzerland), applying the multi-linear reference tissue model with two parameters (MRTM2) [38] and the cerebellar cortex as a reference region, whereas BP_ND_ is defined as (V_*T*_ − V_ND_)/V_ND_ = (V_*T*_/V_ND_) – 1 = k_3_/k_4_. BP_ND_ refers to the specifically bound tracer uptake compared to the non-displaceable uptake at equilibrium. No differences for k2’, the reference tissue clearance rate constant for MRTM2 were found (neutral 1: 0.14 ± 0.04 min^−1^, pleasant: 0.15 ± 0.03 min^−1^, neutral 2: 0.15 ± 0.03 min^−1^, unpleasant: 0.16 ± 0.03 min^−1^, ANOVA *p* = 0.70).

Two datasets were excluded from analysis due to (1) an error with the pneumatic injection system and (2) an error with the PET-fMRI machine. Spatial normalization using the DARTEL approach [39] applied the T1-weighted MPRAGE parameters to the parametric BP_ND_ images, which were then smoothed (8 mm full-width at half-maximum (FWHM) Gaussian kernel). A striatal region of interest (ROI) mask (head of caudate, putamen and nucleus accumbens) was created using the Harvard–Oxford anatomical atlas included in FSL [40] and the mean of the BP_ND_ was extracted. A hypothesis-driven region-of-interest (ROI) approach was adopted based on strong a priori evidence implicating striatal regions [7, 12] in dopaminergic signaling, music-evoked arousal, and corticostriatal interactions. The striatum was selected for PET analyses due to its high D1 receptor density and favorable signal-to-noise characteristics for reliable [^11^C]SCH23390 quantification. Using an explorative analysis, we also applied D1R whole-brain analyses to assess differences between pleasant and unpleasant conditions within the entire brain using SPM (applying an uncorrected peak threshold of *p* < 0.001 with an additional cluster extent of at least 50 voxels).

During fMRI, participants kept their eyes closed but stayed awake. 1200 EPI volumes (i.e. 40 min scanning time) were acquired at a voxel size 2 × 2 × 2 mm^3^, repetition time [TR] 2000 ms, echo time [TE] 30 ms, slice thickness [ST] 3 mm. Data were analyzed in SPM8: images were slice time corrected, realigned, spatially normalized to the Montreal Neurological Institute (MNI) space, and smoothed (8 mm FWHM Gaussian kernel). Time series were band-pass filtered (1/3–1/80 Hz) and detrended with the LIPSIA package (Max Planck Institute for Human Cognitive and Brain Sciences, Leipzig, Germany).

For the single subject level, the first 5 min of images were used in a general linear model with one regressor representing the scans acquired over time. The striatal ROI mask (used for BP_ND_) was used to extract the first Eigenvariate of the beta values. This striatal time series was used as an additional non-interacting regressor in the same model to test for positive correlation (i.e., strengthened functional connectivity) of the striatal seed region throughout the brain. The individual statistic maps, representing the striatal connectivity, were then used in a group-level two-sampled t-test with corresponding individual BP_ND_ values to assess functional connectivity differences between (1) pleasant and unpleasant music stimuli and (2) neutral condition, in relation to the individual striatal BP_ND_ (i.e., fMRI and BP_ND_ interaction). A peak threshold at *p* < 0.001 (uncorrected) and a cluster extent of k > 50 voxel was applied. High test–retest reliability was previously reported for D1R measurement in neutral conditions [41].

### Statistics

Statistical analysis was performed in MATLAB (The MathWorks Inc., Natick, MA, USA). The data were found to be normally distributed using a Shapiro–Wilk test (*p* > 0.05). Paired t-tests were used for group comparison of pleasantness rating and striatal BP_ND_. To compare neutral and music (independent of pleasantness), the mean BP_ND_ of both scans per condition (music was measured during pleasant and unpleasant conditions, silence was measured twice) was calculated. Correlations were assessed using the Pearson correlation coefficient. All analyses were complemented by nonparametric bootstrap resampling (10,000 iterations) to assess robustness independently of distributional assumptions and its bias-corrected and accelerated confidence intervals are reported. The effect size was calculated using Cohen’s *d*. Significance was assumed at *p* < 0.05.

## Results

Pleasant music was rated significantly higher than the unpleasant counterpart (83.5 ± 13.6 vs 30.5 ± 22.3; t(14) = 9.3, *p* = 2.3 × 10^–7^, CI 40.20—62.33, *d* = 2.39).

**Fig. 1:**
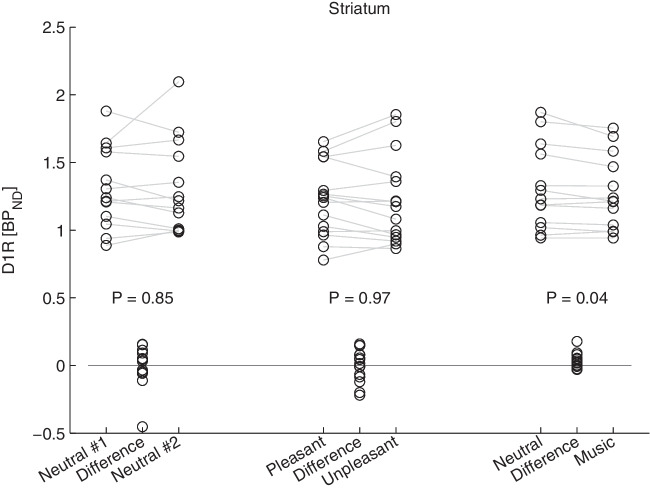
Distribution of striatal BP_ND_ for both neutral scans (left), both scans with pleasant and unpleasant music stimulus (middle) and scans with and without musical stimulus (right). The individual differences between both corresponding scans are plotted in-between with the P value of the corresponding paired t-test shown above

Striatal BP_ND_ during music listening (pooled pleasant/unpleasant) was significantly lower than in the neutral condition (1.27 ± 0.27 vs. 1.31 ± 0.31; *n* = 13; t(12) = 2.3, *p* = 0.04, CI 0.0127—0.0761, *d* = 0.64). No significant difference was found between neutral conditions (1.31 ± 0.3 vs 1.32 ± 0.35; *n* = 13; t(12) = −0.2, *p* = 0.85, CI −0.1311—0.0523, *d* = 0.05) or between pleasant and unpleasant music BP_ND_ (1.22 ± 0.27 vs 1.22 ± 0.33; *n* = 15; t(14) = 0.04, *p* = 0.97, CI −0.0625—0.0549, *d* = 0.01; Fig. 1). The pooled music condition (*n* = 13) yielded a higher mean striatal BP_ND_ than the mean calculated across all music scans (*n* = 15) due to the exclusion of two participants with missing neutral scans and comparatively low BP_ND_ values.

**Fig. 2:**
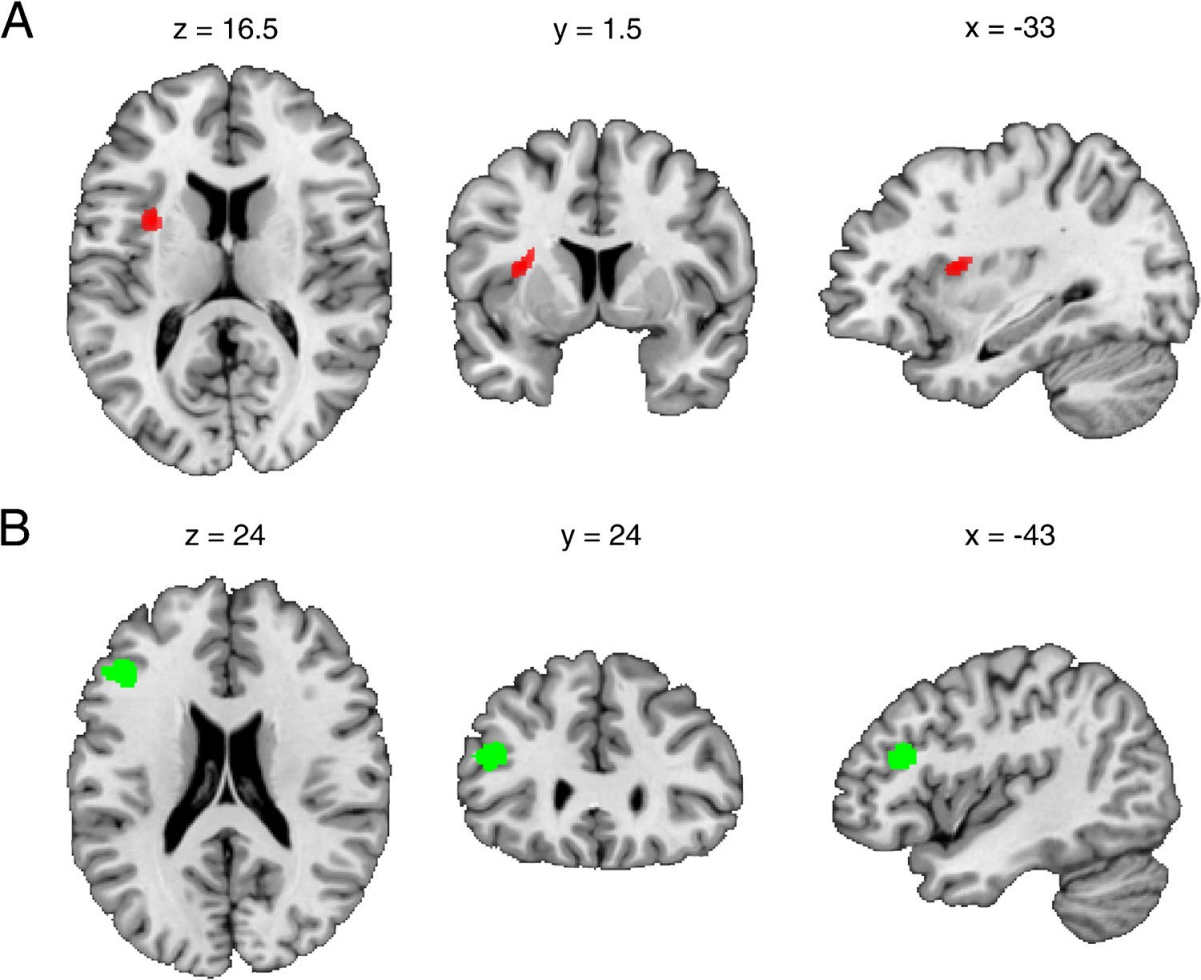
(**A**) Whole-brain D1R PET analysis (*P* < 0.001 uncorrected, k > 50 voxel) showed a significant cluster in the insula (red overlay) with higher BP_ND_ for pleasant music when compared to unpleasant music. The MNI coordinates indicate the location of the peak voxel (T = 5.58; *p* = 3.4 × 10^–5^ uncorrected; k_E_ = 165 voxels). (**B**) The seed-based fMRI analysis (*P* < 0.001 uncorrected, k > 50 voxel) reveals a higher functional connectivity from the striatum (seed) to the left dorsolateral prefrontal cortex (green overlay) when listening to pleasant music compared to unpleasant music in relation to the individual striatal BP_ND_. The MNI coordinates indicate the location of the peak voxel (T = 7.01, P_FWE_ = 0.013)

Whole-brain D1R PET analysis revealed an exploratory cluster in the insula (MNI: x = −33, y = 1.5, z = 16.5; T = 5.58; *p* = 3.4 × 10^–5^ uncorrected; k_E_ = 165 voxels) showing higher BP_ND_ for pleasant compared to unpleasant music (Fig. 2A).

**Fig. 3:**
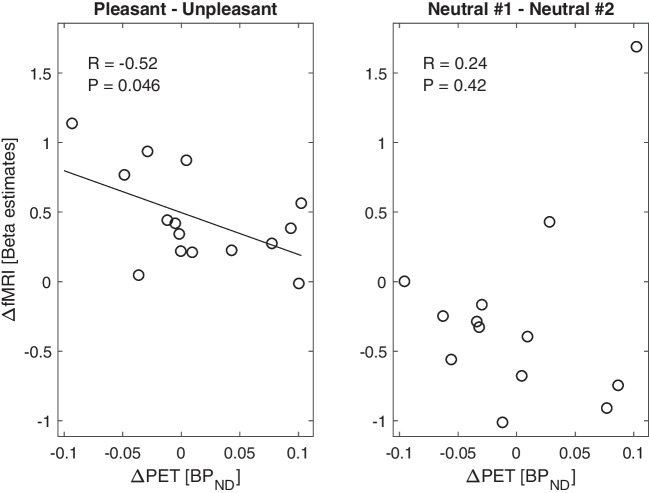
Scatter plots show the distribution of changes in BP_ND_ (PET) and changes in beta estimates (fMRI) within the left dorsolateral prefrontal cluster for music (left) and neutral (right) condition. There is a significant correlation for the changes between pleasant and unpleasant music (left), but not for changes between both neutral scans (right)

**Fig. 4:**
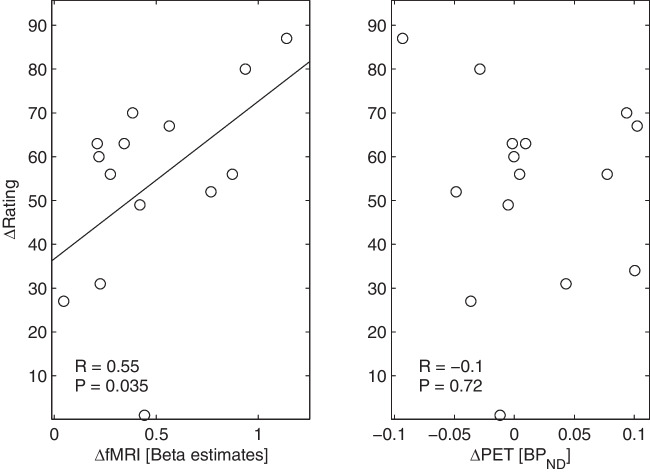
Scatter plots show the distribution of changes in pleasantness rating and beta estimates (fMRI, left) or BP_ND_ (PET, right) within the left dorsolateral prefrontal cluster for music condition. There is a significant correlation for the changes between rating and fMRI (left), but not for changes between rating and PET (right)

In the seed-based fMRI analysis (corrected for multiple comparisons by family-wise error (FWE)), the striatum showed higher functional connectivity (T = 7.01; p_FWE_ = 0.013) with the left dorsolateral prefrontal cortex (MNI: x = −43, y = 24, z = 24) for pleasant music vs. unpleasant music (Fig. 2B), in relation to the individual striatal BP_ND_. No difference in functional connectivity was found between neutral conditions. A moderate negative correlation was found between PET (BP_ND_) and fMRI (beta estimates) changes within that prefrontal cluster for the pleasant vs. unpleasant music (R = −0.52, *n* = 15, *p* = 0.046), but not for neutral conditions (R = 0.24, *n* = 13, *p* = 0.42; Fig. 3). Changes in musical pleasantness ratings correlated moderately with fMRI (beta estimates) changes in this prefrontal cluster (R = 0.55, *n* = 15, *p* = 0.035), but not with PET (BP_ND_) changes (R = −0.1, *n* = 15, *p* = 0.72; Fig. 4). However, nonparametric bootstrap resampling indicated that confidence intervals for all correlations included zero, suggesting that these associations should be interpreted as exploratory.

## Discussion

### The effects of pleasant and unpleasant music on D1R availability

The present findings indicate that striatal D1 receptor engagement during music listening occurs independently of subjective valence. This extends previous work linking dopaminergic release primarily to peak pleasurable moments by suggesting that dopaminergic signaling may also reflect more general features of music perception, such as temporal structure, anticipation, and predictive processing, rather than intense reward experiences alone.

Recent research has shown the involvement of the D1R system in modulating arousal [42, 43]. Our study suggests that music, as a widely accessible and non-invasive tool, offers a promising way to investigate this aspect of the dopaminergic system. Although we did not examine which specific musical features exert the strongest influence, the inherently phasic structure of music likely promotes D1R activation, given the receptor’s sensitivity to phasic dopaminergic input. The instrumental music used here varied over time rather than being strictly repetitive, likely maintaining listener interest and enhancing the observed neural response. Taken together, these findings indicate that, music provides a practical and effective means to probe D1R function.

Previous reports have shown that the use of [^11^C]SCH23390 may be challenging in experimental paradigms investigating D1R function. Thus, increased or decreased endogenous DA through pharmacological interventions didn’t change binding of [^11^C]SCH23390 in rats [44] and non-human primates [45]. On the other hand, Karlsson et al. showed a modification of striatal D1R binding through the cognitive activity of young persons and assumed a relation to endogenous DA release [46]. Moreover, electrically stimulated endogenous DA release in superfused striatal slices of rats inhibited the binding of [^3^H]SCH23390 to D1 receptorssystem [47]. Cognitive training of working memory reduced D1R BP_ND_ of [^11^C]SCH23390, likely reflecting long-term adaptations to sustained increases in endogenous DA during cognitive training [18]. There is also evidence for an inverted U-shaped relationship between prefrontal D1R mediated signaling and working memory performance, indicating that both insufficient and excessive dopaminergic activity can impair executive function [48]. Our results provide additional evidence that [^11^C]SCH23390 can detect subtle changes in D1R BP_ND_. This interpretation is consistent with experimental work demonstrating differential effects of phasic and tonic dopamine release on receptor activation, with D1 receptors being particularly sensitive to phasic dopamine transients [49]. The present findings should therefore be interpreted in terms of D1R-related phasic dopaminergic engagement rather than hedonic pleasure per se, which has been more directly linked to μ-opioid receptor signaling in previous music studies.

Although [^11^C]SCH23390 has been widely used to study the D1R system [48, 50], it also shows affinity for 5-HT2 and 5-HT₁C/5-HT₂C serotonin receptors [15, 17, 51]. However, serotonin-related effects occur only at doses more than tenfold higher than those required to elicit D1R-mediated responses [17, 52]. Thus, while the observed effects are likely D1R-driven, a partial contribution of the serotonin system, which is also involved in arousal, cannot be fully excluded.

### Musical pleasantness initiates further processing mechanism

No musical valence-specific changes in BP_ND_ were observed with PET. Because the tracer applied in this study is not restricted to measuring BP_ND_ changes in the striatum alone, PET-fMRI interactions could be examined across the cortex. Analyzing the fMRI signals acquired simultaneously with PET-fMRI, a valence‐specific effect in the fronto‐striatal connectivity could be observed that depended on the individual striatal D1R availability. To evaluate this, we performed a connectivity analysis to study functional interactions between brain regions during passive listening to pleasant and unpleasant music. In this seed-based fMRI analysis, we found a significantly stronger functional connectivity between the striatum and the left dorsolateral prefrontal cortex (dlPFC) when participants listened to pleasant music compared to unpleasant music. This left-lateralized effect may correspond to earlier theories proposing a dominance of the left hemisphere in processing positive emotions [53]. Within this prefrontal cluster, PET (BP_ND_) and fMRI (beta estimate) changes correlated across the pleasant and unpleasant music conditions. This exploratory association suggests that the observed fronto-striatal interaction may relate to a D1R-related mechanism that is particularly engaged during pleasant music listening. However, it should be noted that the observed associations between striatal D1R binding and fronto-striatal functional connectivity are correlational in nature and do not allow causal inferences regarding D1R-mediated modulation of network dynamics. The PFC is frequently discussed as part of the dopaminergic network, and stimulation of the PFC with repetitive transcranial magnetic stimulation (rTMS) has been shown to induce endogenous dopamine release in the ipsilateral dorsal caudate nucleus, as demonstrated with PET using [^11^C]raclopride [54]. Furthermore, the PFC has been implicated in a large variety of studies examining brain responses to music (e.g. [30, 55–58]). The dorsolateral part of the PFC (dlPFC) is known for its crucial role in working memory [59], and lesion studies showed that the left dlPFC, in particular, is necessary for manipulating verbal and auditory information in working memory [60]. In the dlPFC, auditory-spacial information appears to be processed, as investigated in non-human primate studies [61–63], and in humans as well [64]. Interactive Jazz improvisation has also been shown to activate the dlPFC in an fMRI study by Donnay et al. [57]. Activity in a similar region (left MNI: x = −42, y = 22, z = 22) was observed in our analysis. Comparable bilateral activation was measured when musically naïve subjects learned to associate musical chords with an arbitrary number [58]. Musicians, especially those with absolute pitch, have been demonstrated to exhibit stronger activation of left posterior dlPFC than non-musicians during passive music listening [65]. Monitoring PFC activity with functional near-infrared spectroscopy (fNIRS) during a free-recall task with background music showed enhanced verbal memory performance [66], suggesting a potential therapeutic role of music for people with memory impairments. In a music memory task, activation of the left inferior frontal gyrus (MNI: x = −45, y = 27, z = 24) was associated with successful retrieval [67]. Our results are consistent with previous studies reporting left-hemispheric lateralization in response to pleasant music, a phenomenon often described within the framework of the `valence lateralization model´ [31, 68, 69]. Exploratory whole-brain PET analyses further revealed a valence-related effect in the left anterior insula, with higher D1R binding during pleasant compared to unpleasant music. Although this effect did not survive correction for multiple comparisons and should therefore be interpreted with caution, it is consistent with previous fMRI findings implicating the anterior insula in music-evoked emotional processing, particularly for pleasant music [32].

Given the role of the PFC in emotion, controlling the release of the mood-altering neurotransmitters DA, serotonin, and norepinephrine, it seems plausible that the functional connectivity effects observed in the current data may relate to a gateway function of the PFC to working memory capacities that is modulated by positive affect (perceived valence of the music) [70, 71].

### Implications for music as a tool in rehabilitation

Music-based interventions may benefit disorders involving dopaminergic dysfunction, including depression, ADHD, schizophrenia, and Parkinson’s disease, potentially via music-induced engagement of D1R signaling that is consistent with reported clinical effects [72–82] and our observation of reduced D1R BP_ND_ during music listening.

### Limitations

Our study compared music to silence, which means that the observed D1R changes may not be music-specific but could reflect responses to acoustic stimulation more generally. The present design does not allow dissociating which specific musical features (e.g., rhythm, tempo, or harmonic complexity) primarily drive D1R engagement during music listening. Although the effects are likely D1R-mediated, a contribution of the serotonin system cannot be entirely ruled out due to tracer properties, even though it is less sensitive to serotonin at the doses used representing a methodological limitation of the present study. Finally, because the silent conditions were acquired in the morning and the music conditions in the afternoon, we cannot exclude an influence of diurnal variation or residual order effects on the observed differences.

A further limitation of the present study is the absence of a temporally structured non-musical control condition, such as phasic noise. The paradigm was intentionally designed to elicit sustained dopaminergic engagement over extended periods, which can be robustly captured using PET with the D1 radioligand [^11^C]SCH23390, rather than brief phasic dopamine transients that are difficult to resolve against baseline dopamine levels using current PET methodology [83, 84]. As a consequence, the present findings cannot determine whether the observed D1R engagement is specific to music or reflects a more general response to temporally structured auditory stimulation. Individual differences in musical preferences and the use of artificially dissonant stimuli may have influenced emotional responses. No explicit partial volume correction was applied, and therefore partial volume effects related to limited spatial resolution may have influenced regional PET signal estimates. A limitation of this proof-of-concept study is the modest sample size and age range, which may limit statistical power and generalizability of the findings.

## Conclusion

This PET-fMRI study with [^11^C]SCH23390 demonstrates that music activates the D1R system, as reflected by significantly lower striatal BP_ND_ during music listening compared with silence. This indicates that the phasically sensitive D1R system can be effectively stimulated by the inherently phasic structure of music, independent of perceived pleasantness, likely driven by musically mediated arousal. These results highlight music as a widely accessible tool for probing D1R function, offering new opportunities for studying disorders involving D1R dysregulation. Replication in larger cohorts will be important to confirm the generalizability of these findings.

## Data Availability

The datasets generated and analysed during the current study are available from the corresponding author on reasonable request. The data are not publicly available due to their containing information that could compromise the privacy of the participants.
